# Combating HIV and/or AIDS: A challenge to Millennium Development Goals for disaster managers in the Southern African Development Community

**DOI:** 10.4102/jamba.v8i2.173

**Published:** 2016-01-13

**Authors:** Olivia Kunguma, Alice Ncube

**Affiliations:** 1 Disaster Management Training and Education Centre for Africa, University of the Free State, South Africa

## Abstract

Disaster management is a process of planning and implementation of measures involving multiple disciplines and sectors; hence Millennium Development Goals (MDGs) cannot go unnoticed. Approximately 189 member states agreed to endeavour to achieve MDGs which should be accomplished by 2015. The purpose of this research was to establish the primary involvement of the disaster management fraternity within the Southern African Development Community (SADC) region in this agreement. SADC countries are the countries with a high prevalence of HIV and AIDS and they feature on the disaster manager’s priority list of hazards, hence the focus on MDG 6 for this study. Various data gathering tools were employed and included making use of indicators developed by the United Nations to review disaster management statutes or civil protection statutes and scholarly documents on the progress of MDG 6. Structured interviews were carried out with heads of disaster management centres of SADC countries through the guidance of MDG 6 indicators. The main findings were that most statutes do acknowledge the fight against epidemics and most disaster managers are aware of MDG 6 and are involved in its achievement. It was recommended that disaster managers should be part of the Post 2015 MDG delegation.

## Introduction

In the year 2000, the heads of 189 countries signed the United Nations Millennium Declaration to commit to the combating of poverty, hunger, disease, illiteracy, environmental degradation and discrimination against women by the year 2015 (United Nations 2010). Disaster management plays a big role within a countries’ government service, of providing humanitarian assistance, and because of the nature of the Millennium Development Goals (MDGs), the disaster management sector is likely to be expected to contribute immensely in the achievement of these goals. The disaster management fraternity encompasses non-governmental, governmental and private sectors which all contribute to humanitarian activities. However, for the purposes of this research, only the government sector will be explored. The focus is only on this sector because the state is responsible for setting up the environment and the legal framework in which its departments and other organisations operate. The government of a country oversees all the activities that are carried out by different organisations: national and international, private and non-profit making organisations, hence the signatories include heads of states. The term Disaster Managers as humanitarian officials is used in this research, however, in different SADC countries these humanitarian officials are given different titles.

According to United Nations Development Program (UNDP) (2013a) the MDG 6 goal aims at combating HIV and AIDS, Malaria and other diseases. The anticipated outcomes of MDG 6, with HIV and AIDS in particular, are amongst the many targets that many countries are struggling to reach. Although the universal access to antiretroviral therapy for all who need it was anticipated to be reached by 2015, it was evident that this target was beyond accomplishment by the year 2010. This is worrisome because of the infection rate in low and middle income countries where approximately 820 000 women and men aged 15–24 were newly infected with HIV in 2011. According to the United Nations (2013), basic knowledge about how to prevent HIV amongst young women and men in Sub-Saharan Africa remains low, as it is a region that is globally hardest hit by this epidemic. Sub-Saharan Africa is the region with the most number of people living with HIV worldwide.

As discussed above, the complex nature of MDG 6, in particular HIV and AIDS, has made it a cause for concern and is of interest to the researchers. As the researchers are in the disaster management profession, they focused on disaster management as a humanitarian fraternity with regards to their involvement in the achievement of this specific goal. This study was undertaken with two objectives, which are as follows:

- To review disaster management statutes of SADC countries, to identify if they support the achievement of the MDG 6 with a focus on HIV and AIDS.
- To determine if the disaster management fraternity within the SADC region recognises the Declaration and the importance of achieving the millennium goals.

The successful achievement of MDG 6 and all the other MDGs requires country ownership and commitment by the government, to spur the institutional changes that are needed to effectively ensure the sustainability of capacity building efforts. This research was strictly undertaken on the scrutiny of the Disaster Manager’s involvement and contributions towards the achievement of MDG 6, with a focus on HIV and AIDS, hence the purposive exclusion of the health statutes review and the Health Ministries of the respective countries.

## Southern African Development Community region

**FIGURE 1:**
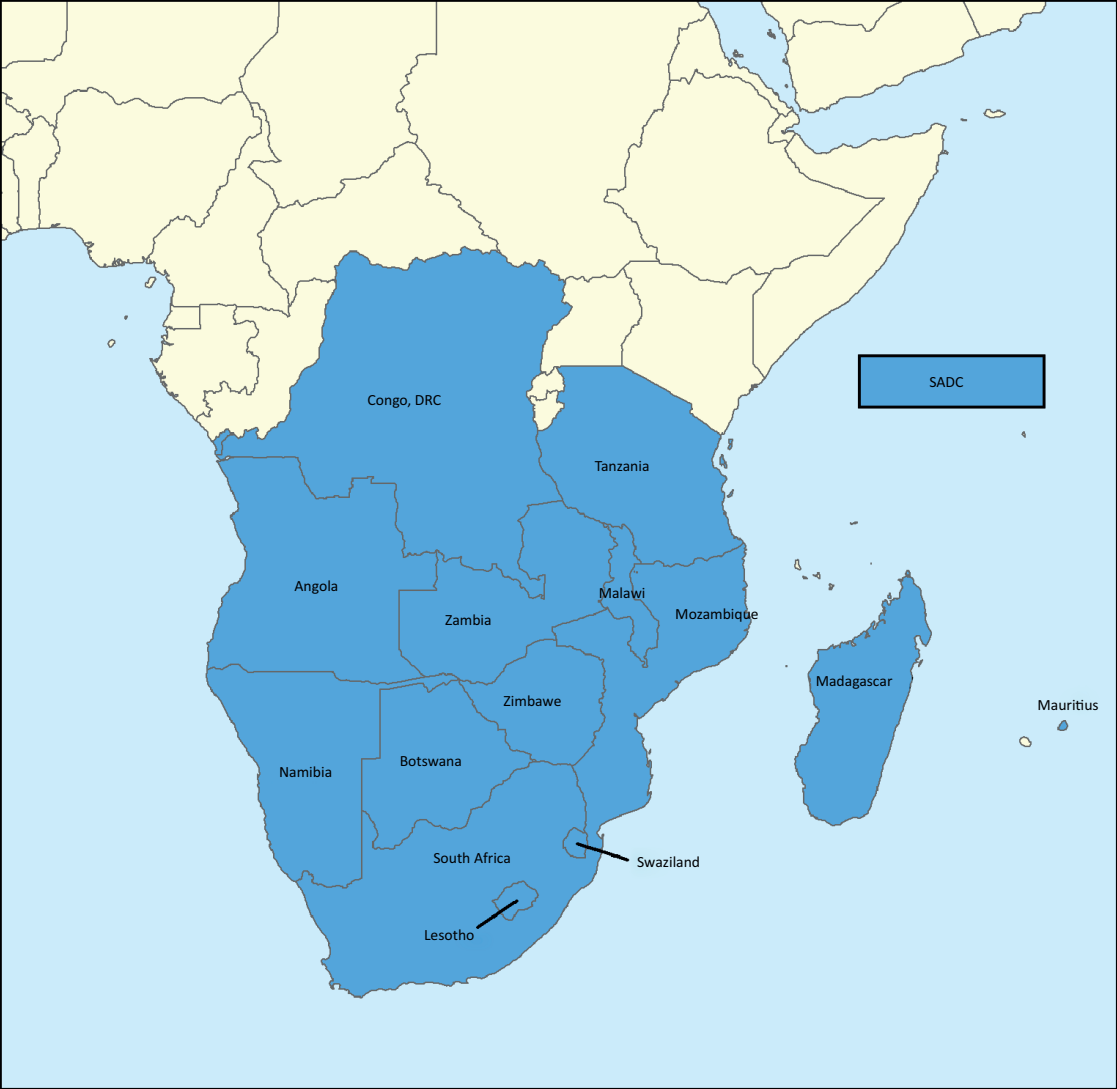
Map of Southern African Development Community member states.

The Southern African Development Coordinating Conference (SADCC) was formed in 1980 (later to become SADC), and functioned as an informal partnership with the main aim of coordinating development projects. Through the Windhoek Treaty of 1992, the SADC replaced the SADCC and became a development community with a legal identity, to promote unity, social and economic integration and development in the region. The SADC consists of 15 Member States and is a relatively large sub-region (6.2 million km²) composed of countries of diverse sizes (SADC 2008). The map in Figure 1 shows the SADC member states.

The SADC, since its inception, has understood the importance of the role that the health sector and health provisions impact on all areas of development in the region. Heads of member states agreed to set up the SADC Protocol of Health of 1999, which became operational in 2004. The protocol recognised HIV and AIDS as major areas of focus for the health sector (SADC 2008).

## Overview of HIV and/or AIDS in Southern African Development Community

According to the UNAIDS Global Report (2013) and (UNDP 2013b) the highest number of HIV and AIDS cases are found in Sub-Saharan Africa. It is estimated that in 2012, of the 25 million that were living with HIV, 70% were to be found in Sub-Saharan Africa. An estimated 1.6 million new infections and 1.2 million AIDS-related deaths were also reported in 2012.

Southern Africa is presently the epicentre of this epidemic, with countries such as Swaziland and South Africa experiencing the highest prevalence of HIV, and highest number of HIV cases respectively. Botswana and Lesotho are not far behind Swaziland in terms of high HIV prevalence rates in the region (Botswana 2010; Lesotho 2006).

The key drivers fuelling the spread of the disease in the region are the widespread practice of polygamous relationships as well as the multiple sex partners that many men have at any given time. The crippling effects of the HIV epidemic have far-reaching social and economic consequences, affecting not only the health sector but every aspect of governance and development in the region. The scenario presented above does not bode well for the region’s ability to meet MDG 6 (UNAIDS 2013).

Table 1 highlights the estimated number of HIV and/or AIDS cases in 2013, in proportion to the population of the SADC countries.

**TABLE 1:** Estimates of HIV cases 2013 (Southern African Development Community region).

Country	Population mid–2013 (millions)	HIV cases
Angola	26.1	250 000 [180 000–340 000]
Botswana	1.9	320 000 [310 000–340 000]
Democratic Republic of the Congo	71.1	440 000 [370 000–520 000]
Lesotho	2.2	360 000 [350 000–380 000]
Malawi	16.3	1 000 000 [970 000–1 100 000]
Mauritius	1.3	9600 [8700–11 000]
Mozambique	24.3	1 600 000 [1 400 000–1 800 000]
Madagascar	22.5	54 000 [46 000–64 000]
Namibia	2.4	250 000 [210 000–290 000]
Seychelles	0.1	No data available
South Africa	53	6 300 000 [6 000 000–6 500 000]
Swaziland	1.2	200 000 [200 000–210 000]
United Republic of Tanzania	49.1	1 400 000 [1 300 000–1 500 000]
Zambia	14.2	1 100 000 [1 100 000–1 200 000]
Zimbabwe	13	1 400 000 [1 300 000–1 400 000]

*Source:* 2013 Population-data-sheet; WHO, 2015, *World health statistics 2015*, viewed 06 June 2015, from http://www.prb.org/pdf13/2013-population-data-sheet_eng.pdf

Table one shows the estimated population for 2013 which was included to bring clarity when the number of HIV cases are examined against the overall population, as well as compared with the population and number of cases in other SADC countries. For example, Botswana has a total population of 1.9 million, but has approximately 320 000 HIV and AIDS cases. This shows that a huge portion of the population is infected with HIV and AIDS. This picture is made worse if you compare it with Angola which has a population of 26.1 million, but only 250 000 HIV and AIDS cases, or with the DRC which has a population of 71.1 million and only 440 000 cases. Mauritius and Swaziland have more or less the same population of 1.3 and 1.2 million respectively but have a huge difference in the number of HIV and AIDS cases, with Mauritius having only 9600 cases and Swaziland 200 000 cases. South Africa has the highest number of HIV and AIDS cases in the SADC region with 6 300 000 million cases in a population of approximately 53 million.

## Research design and methodology

The research made use of a qualitative approach because of its exploratory and interpretive nature. The content of relevant literature was systematically examined in detail for the purpose of identifying specific themes and biases which influence the support of MDGs by the disaster management public sector within the SADC countries. For the research design and methodology of the study, various data gathering tools were employed. This included the use of indicators developed by the United Nations to monitor and evaluate the progress of the set targets that have been imposed with the goal of combating HIV and AIDS. MDG 6 has three main targets but this research focuses on only two target areas and the indicators are listed in Table 2. The indicators were used in this study to analyse the achievement of SADC countries in reaching these targets, and to analyse if disaster managers in each country made any contribution. The indicators were also used in the telephone interview questionnaire asked to heads of SADC national disaster management centres.

**TABLE 2:** Millennium development goal 6 indicators.

Target	Goal	Indicators
Target 6.A	Have halted by 2015 and begun to reverse the spread of HIV and/or AIDS	HIV prevalence amongst population aged 15–24 years Condom use Proportion of population aged 15–24 years with comprehensive correct knowledge of HIV and/or AIDS Ratio of school attendance of orphans to school attendance of non-orphans aged 10–14 years
Target 6.B	Achieve, by 2010, universal access to treatment for HIV and/or AIDS for all those who need it	Proportion of population with advanced HIV infection with access to antiretroviral drugs

*Source:* Adapted from Millennium Development Goals Indicators: The official United Nations site for the MDG Indicators 2008, viewed 08 July 2014, from http://mdgs.un.org/unsd/mdg/Host.aspx?Content=indicators/officiallist.htm

Heads of the Disaster Management centres for the 15 member states were contacted telephonically for their input. Contact details were collected from the United Nations (UN) website for the individual countries. Questions for the telephone interviews were compiled according to the indicators for MDG 6. During the telephone interviews the heads of the centres were interviewed to determine their knowledge of each target, and in which indicators they were involved or made a contribution. Document analysis and review formed part of the methodology with Disaster management statutes of all the SADC countries being collected and reviewed in detail to examine if they support the MDG 6. Most of the documents analysed were downloaded from the websites of the various countries. The key terms that were focused on were not limited to health related issues, HIV and AIDS and epidemics. Other documents relating to MDG 6 were reviewed to ascertain the measures taken by the SADC’s disaster managers to achieve this goal. The findings were presented in a narrative form under each methodology and objective.

## Limitations of the study

Reviewing and obtaining the statues was restrictive because not all countries have their documents loaded and accessible on the internet. Additionally, some of the documents obtained were either in French or Portuguese. However, the documents were loaded onto Google translate but the language translations proved difficult to follow. Despite this, 9 out of 15 countries statutes were reviewed, which represents 60% of the SADC countries. Obtaining working telephone numbers for SADC disaster management centres was also an obstacle and, furthermore, holding an interview with some of the officials could not be accomplished. Questionnaires were also emailed which resulted in a limited response. Some of the officials were not willing to discuss anything despite the assurance that the research is for academic purposes only, thus, questionnaire responses were limited.

## Strategies implemented to achieve Millennium Development Goal 6 targets

There are strategies that have been put in place to avert HIV and AIDS. According to the United Nations (2013), condoms are one of the most efficient means available to help prevent and mitigate the sexual transmission of HIV. This is followed by abstinence, being faithful to one partner and antiretroviral treatment (ATT). It is then imperative to investigate which strategies disaster managers have contributed towards, or implemented as a strategic entity. These will be discussed under interviews carried out with national disaster management heads.

## Southern African Development Community disaster managers: Milestones in achieving Millennium Development Goal 6

Table 3 shows how far SADC countries have come as far as reducing the incidence of HIV and AIDS from 2001–2013.

**TABLE 3:** Percent reduction of HIV incidence 2001–2013 (Southern African Development Community region).

Country	% reduction in HIV incidence, 2001–2013
Angola	-8
Botswana	70
Democratic Republic of the Congo	51
Lesotho	25
Malawi	80
Mauritius	69
Mozambique	48
Madagascar	66
Namibia	61
Seychelles	No data available
South Africa	57
Swaziland	48
United Republic of Tanzania	67
Zambia	62
Zimbabwe	56

*Source:* WHO, 2015, *World health statistics 2015*, viewed 06 June 2015, from http://www.prb.org/pdf13/2013-population-data-sheet_eng.pdf *Note:* Target 6.A, have halted by 2015 and begun to reverse the spread of HIV and/or AIDS.

According to World Health Organization (WHO) (2014), the SADC region remains the epicentre of HIV and AIDS. The prevalence of HIV remains above 10% with no signs of abating. It is further stated that the chances of halting this scourge by 2015 are becoming slimmer (WHO 2014). Whilst other countries have made great strides to overcome HIV and AIDS, some are still struggling with the epidemic. Angola, for example, has made no progress at all in terms of reducing the incidence of HIV and AIDS, experiencing a negative percentage reduction of -8%. Lesotho has only seen a progress of 25%, with Mozambique and Swaziland at 48%. Botswana achieved a 70% HIV and AIDS incidence reduction resulting from the efforts of the government of Botswana and other humanitarian organisations, including HIV prevention and mitigation, reducing the number of annual AIDS deaths and encouraging the increase of voluntary HIV testing and counselling services. Malawi is listed amongst the top 20 African countries that are making great progress in achieving this goal and consistently reducing HIV prevalence. The table above shows that Malawi has made huge progress, achieving an 80% reduction in HIV incidence since 2001. Additionally, there has been a notable decline in new HIV infections in Mauritius, at 69%.

According to African Health Observatory (2014) and Mozambique (2010), as a result of the strong commitment of the government of Mozambique to the fight against HIV and AIDS, the country has approved important instruments that are used as a guide to design actions to fight this disease at national level, namely the Prevention Acceleration Strategy (2008), the National Strategic Plan III (2010) and the implementation of the Nutritional Support Strategy for People Living with HI/AIDS and other chronic diseases (2007) It was through the implementation of the second medium term Strategic Plan for Tuberculosis and Leprosy 2010–2015, which takes into account TB associated with HIV and AIDS and leprosy at national level. This plan resulted in the reduction of the HIV and AIDS incidence in Namibia surpassing the 2015 targets (UNDP 2013b) In South Africa there are some improvements in the efforts to achieve MDG 6. According to the table above South Africa achieved an HIV and AIDS incidence reduction of 57%. Furthermore, the South African Country Report (UNDP 2013b), states that HIV prevalence amongst the age group of 15–24 years has gone down by almost 10%, thus indicating that 2015 targets have been achieved (2013b).

## Content analysis: Southern African Development Community countries disaster management statutes

This section of the study discusses the findings obtained from the review of disaster management statutes of SADC countries, to examine if they support the achievement of the MDG 6. The documents obtained were labelled differently, as: policies, decrees, acts, frameworks, reports, plans and bills. Table 4 illustrates the list of countries and the collected statutes for each country.

**TABLE 4:** List of Southern African Development Community countries and statutes used for disaster management or civil protection.

Countries	Statute(s)
Angola	National plan for preparedness, Contingency, Response and Recovery to Calamities and Natural Disasters Plan for 2009–2014 (Presidential Decree No.205/10)
Botswana	National Policy on Disaster Management, Presidential Directive No. CAB. 27/96. 1996 (Botswana 1996)
Democratic Republic of Congo	Zaire: Ministerial order establishing and organising a national program of prevention and fight against natural disasters - PRONAPLUCAN (Ministerial Order no. CAB/VPM/AS/0016199/91 of 16 August 1991) (Democratic Republic of Congo 1991)
Lesotho	*Disaster Management Act* (No.2 of 1997)
Madagascar	Madagascar: *Décret fixant les modalités d’application de la Loi no. 2003–010 du 5 septembre 2003 relative à la politique nationale de gestion des risques et des catastrophes (Décret no. 2005–866 du 20 décembre 2005)* (Madagascar 2005)
Malawi	*Disaster Preparedness and Relief Act* (No. 24 of 17 December 1991)
Mauritius	*No document found*
Mozambique	*Mozambique: Plano director para prevenção e mitigação das calamidades* (Mozambique 2005)
Namibia	*Disaster Risk Management Act* (No. 10 of 2012)
Seychelles	Under review
South Africa	*Disaster Management Act* (57 of 2002)
	Disaster Management Framework of 2005
Swaziland	*Disaster Management Act* (2006)
Tanzania	*Disaster Relief Coordination Act* (No.9 of 1990)
Zambia	*Disaster Management Act* (No. 13 of 2010) (Zambia 2010)
Zimbabwe	*Civil Protection Act* (5 of 1989)

Each statute was analysed in detail for any mention of HIV and AIDS and MDGs. This was presented in Table 5. Any mentioning of any of these words in the statutes is assumed to be a possible state of readiness for dealing with MDG 6 through the existence of clinics, Paramedic and Health Ministries. MDGs are an international initiative which require national legal documents to support the initiative and share the same goals.

**TABLE 5:** Words mentioned in the statutes analysed.

Country	Number of statutes	HIV and/or AIDS	Health and institutions	Epidemic or virus or disease or other
Angola	-	-	-	-
Botswana	1	-	Ministry of Health	Epidemics
Democratic Republic of Congo	-	-	-	-
Lesotho	1	-	Health and Nutrition Group, Secretary: Health and Social Welfare, District Medical Officer, Medical Supplies	-
Madagascar	-	-	-	-
Malawi	1	HIV and/or AIDS	Health sector, Secretary: Health and Population, Medical Supplies	Plague, Epidemic, Disease
Mauritius	-	-	-	-
Mozambique	-	-	-	-
Namibia	1	-	Nurses, Health and Social Services, World Health Organization	Plague, Epidemic, Disease
Seychelles	-	-	-	-
South Africa	2	-	Clinics, Paramedics, Emergency Medical Services, Environmental Health	Epidemics
Tanzania	1	-	Health care	Epidemics
Swaziland	1	HIV and/or AIDS	Health and Social Welfare, Medical, Paramedical	-
Zambia	1	-	Health Care, Health Practitioner, Health Facility, *Health Professions Act*, 2009	-
Zimbabwe	1	-	Secretary for Health, Medical supplies	Plague, Epidemic, Disease

The disaster management statute of each SADC country is discussed in relation to the MDG 6, its recognition of health issues, support of the involvement of different disciplines and sectors in preventing and mitigating the impact of diseases are also taken into consideration.

### Angola

According to IFRCS (2012) there are various legal documents for Disaster Management operations in Angola, however, there is no standalone document. Disaster management activities are mainly addressed by a National Plan of Preparation, Contingency, Response and Recovering from Calamities and Natural Disasters (NPPCRRCND). These documents could not be found on the internet for analysis.

### Botswana

The policy recognises and supports active participation in international conducts like the International Decade for Natural Disaster Reduction (IDNDR). The policy recognises the Ministry of Health as the focal point for dealing with epidemics. Identification and recognition of these national and international organisations is a positive indication for fighting and mitigating the impacts of diseases as well as possible support for MDGs (Botswana 2010).

### Democratic Republic of Congo, Mozambique and Madagascar

In the DRC the only document that was found, was last developed when the DRC was still named Zaire. Unfortunately this document is in French and could not be interpreted. According to Dr J.T. Lukusa, a Congolese disaster management student, there is no whole Act for Disaster Management but each relevant ministry has a section of disaster management. The Ministries responsible for Disaster Management are:

- Ministry of Defence and National Security
- Ministry of Environment and Natural Resources
- Ministry of Public Health
- Ministry of Humanitarian and NGOs.

The Mozambique document is in Portuguese, however, valuable information was gathered during a telephone interview. Madagascar’s statutes were not found.

### Lesotho

Lesotho is one of the first countries in the SADC region to have its leader, His Majesty King Letsie III declare HIV and AIDS a national disaster, in the year 2000. This came about after Lesotho was one of the countries, internationally, with the highest HIV and AIDS infection and prevalence rates. This declaration was made at the same time that the MDGs were established, which was a perfect occasion for the injection of any resources needed to address the HIV and AIDS scourge. Within the *Lesotho Act* (2 of 1997), there is a National Disaster Relief Task Force and the Health and Nutrition Group forms part of this task force. This recognition by disaster management shows a possibly effective contribution to MDG 6 attainment (UNDP 2011).

### Malawi

The Department of Disaster Management Affairs (DDMA) supports the assistance of international organisations like the UN and the UNDP in pursuit of achieving the MDGs. HIV and AIDS is mentioned in the Act (24 of 1991) as a cross cutting issue, which makes it an important area of concern for the Malawi government. Plagues, epidemics and diseases are mentioned in the definition of disaster, as possible occurrences that might threaten life or the wellbeing of a community. Additionally, the Secretary for Health and Population sits on the National Disaster Preparedness and Relief Committee of Malawi.

### Mauritius

Only a bill, cited as the *Mauritius Fire and Rescue Service Act* of 2013, was found on the internet. This could be an indication that Mauritius is still reactive rather than proactive about this epidemic, or their Act was not posted online.

### Namibia

In its definition of disaster, plaques, epidemics and diseases, these are identified as possible events that might threaten a community’s health and thereby take precedence over other circumstances. In addition to this the National Disaster Risk Management Committee has the Ministry of Health and Social Services as one of the advisors to the President and Cabinet. There is the possibility that this Ministry also advises and updates the President about the advancement in MDG 6. The Namibian Vulnerability Assessment Committee, constituencies and sub-committees have representatives for health emergency management and nurses who are usually the primary health care providers and best advocates for fighting diseases. Furthermore, according to Griffiths and Maben (2008) nurses are crucial to the delivery of 21st century healthcare. Another positive aspect of the *Disaster Risk Management Act* (10 of 2012) is the support and participation of organisations such as the WHO for health issues in Namibia.

### Seychelles

According to Ms Divina Sabino, the head of Risk and Disaster Management which is a division within the Ministry of Environment and Energy, the disaster management policy is currently being updated and has been submitted to the cabinet for endorsement.

### Tanzania

In the *Disaster Relief Coordination Act* (9 of 1990), under the definition of disaster, medical care is mentioned as a need that should be provided. This is a possible indication that MDG 6 is provided for by the disaster management fraternity.

### Swaziland

It is stated in the Act (1 of 2006) that the Minister in charge of disaster management shall ensure integrated systems and structures for HIV and AIDS management in Swaziland and this stands as a positive initiative for achieving MDG 6. To show its support for attaining MDG 6, the Act has on its committee the Health and Social Welfare portfolio.

### South Africa

The *Disaster Management Act* (57 of 2002) and National Disaster Management Framework (2005) include a rich referral to the words sought after. Health institutions are mentioned as some of the portfolios that should sit in advisory forum meetings, such as the International Committee of Disaster Management (ICDM) and the National Disaster Management Advisory Forum (NDMAF), and other forums at different spheres of the South African government. Specifically in the Disaster Management Framework of 2005, under Key Performance Area 1, on integrated institutional capacity for disaster risk management, it is stated that an effective and comprehensive disaster risk management strategy cannot be achieved without participative decision making, involving a wide range of role players. It also states that it is imperative, for disaster risk management in South Africa, to be informed by a global perspective so that it remains at the cutting edge of developments. It must associate itself with selected international development protocols, agendas and commitments, such as the millennium development goals outlined in the UN Millennium Declaration. Having such guidance in the National Framework is a strong indication of the South African Governments participation in achieving the goals of the MDGs. The Framework further states that the National Disaster Management Centre (NDMC) should forge links with national agencies such as the WHO and the Joint United Nations Programme on HIV and AIDS (UNAIDS). To keep abreast of international developments, the framework has key performance indicators, and one relevant indicator specific to this study is that a disaster risk management forum must be established for the purpose of co-operation with countries in the SADC region, for effective operation. South Africa’s commitment to contributing to and achieving MDG 6 is found in these two statutes.

### Zambia

In the respective Act (13 of 2010), health care is mentioned as an essential commodity, which is a positive recognition when it comes to dealing with any diseases that might threaten Zambia. Epidemics are listed as potential hazards that might affect the country, and this is a form of preparedness. For the successful achievement of MDG 6, all relevant experts needed to avert epidemics are required to be involved on the Zambian National Disaster Management Council (Zambia 2011). The Ministry of Health forms part of this council. Records showing the location of health facilities and the particulars of health practitioners are mandated to be included.

### Zimbabwe

Epidemics, plagues and diseases form part of the country’s definition of disaster. This is a positive perspective. There is also a National Civil Protection Committee mentioned in this definition, of which the Secretary for Health should be a member. This reinforces the government’s commitment to disaster relief. Whilst a declaration of a state of disaster is in force, the civil protection officer may, by order in writing, direct any person to maintain specified stocks of medical supplies and other supplies for use during the disaster. This is a positive section in the *Civil Protection Act* (5 of 1989) which indicates support for the forestalling of any disease and the possible contribution to the achievement of MDG 6, given the availability and accessibility of resources in the country. However, this Act does not show any support or mention of any international arrangements.

## Interviews with heads of national disaster management centres

Table 6 illustrates the countries which were contacted and the various responses encountered. From the 15 SADC countries all disaster management centres were contacted via telephone numbers obtained from the UNDP website. However, there was no answer from Madagascar, Tanzania or Angola. The number listed for Mauritius was for the Weather Services Department and, hence, there was no success. For the DRC, Botswana, Namibia, Zambia, Lesotho and Swaziland, the respondents requested the questionnaire to be emailed to them, but it was never returned. Numerous follow-up phone calls were made to no avail. Below are the successful responses from South Africa, Zimbabwe, the Seychelles, Malawi and Mozambique.

**TABLE 6:** Interviews with heads of national disaster centres.

Countries	Responses
Angola	No answer
Botswana	Requested questionnaire to be emailed
Democratic Republic of Congo	Requested questionnaire to be emailed
Lesotho	Requested questionnaire to be emailed
Madagascar	No answer
Malawi	Telephonic interviews
Mauritius	Wrong number listed
Mozambique	Telephonic interviews
Namibia	Requested questionnaire to be emailed
Seychelles	Telephonic interviews
South Africa	Telephonic interviews
Swaziland	Requested questionnaire to be emailed
Tanzania	No answer
Zambia	Requested questionnaire to be emailed
Zimbabwe	Telephonic interviews

*Source:* SADC 15 member states, viewed from http://ec.europa.eu/eurostat/web/international-statistical-cooperation/africa-caribbean-pacific/africa-sub-saharan/east-and-southern-africa

From the telephone interviews conducted with heads of departments or representatives designated by their heads, the responses were categorised into the following five findings:

- knowledge of MDG 6
- strategies and projects implemented or involved in
- successes and challenges encountered with halting MDG6
- other diseases being dealt with
- countries rating of the achievement of MDG 6 before 2015.

Mozambique: Ms S. Chilengue, Head of training, gender and capacity building (Institute for Disaster Management Mozambique), as a representative of the Institute for Disaster Management Mozambique (IDMM), stated that she was aware of MDG 6 and what it entailed. The institute is working with various national and international organisations to combat diseases, for example UNICEF and OXFAM. They conduct awareness campaigns together with these organisations in schools and communities at large, about HIV and AIDS. Success is indicated in their work with other organisations and that they attended a meeting hosted by OXFAM a week earlier than the date of this interview, which had MDGs on their agenda. They also carry out vulnerability assessments on a yearly basis. The challenges faced include strong cultural beliefs embedded in the perspective of most people. One striking issue is that of non-belief in the use of condoms. Not everyone believes in condom usage. Other challenges are gender biases and the lack of financial capital for women, which results in their vulnerability and exposure to dangerous lifestyle options, resulting in them being infected with HIV. Mozambique rated themselves as high in terms of MDG achievement, resulting from the assistance received from donors and monitoring and evaluation programs (Telephone interview, 21 July 2014).

Malawi: In his questionnaire response, Mr James Chiusiwa, a representative of Malawi DDMA, displayed his knowledge about MDGs. With regard to strategies applied by DDMA to address MDG 6, they have partnered with the United Nations Population Fund (UNFPA) to ensure delivery of high quality health services in times of emergencies, using a Minimum Initial Service Package (MISP), which also focuses on sexual and reproduction health. MDGs have also been incorporated in DDMA’s contingency plans at both district and national level. With the Malawi’s Disaster Risk Management Institutional Structure, there is a technical subcommittee that focuses on health issues. In their attempt to achieve this goal, the contingency plans entail a component on disease outbreaks which focuses on prevention. As part of the prevention program during disasters or emergencies DDMA ensures availability of condoms and other reproductive health services through the assistance of UNFPA.

Zimbabwe: Ms Sibusiso Ndlovu, the representative for Civil Protection Department Zimbabwe, explained the following in an email response:

It would not be meaningful for us to complete this questionnaire; Coordination of achieving MDGs is under an inter-ministerial committee set up specifically for that task. Our mandate for comprehensive DRM in the country is not fully realised as requisite policy and legislation remains under consideration. MDGs are largely sectoral and coordinated by a specific inter-ministerial committee. We are fully conversant on what MDGs are and the progress or lack of it. (n.p.)

The interview questionnaire was disregarded in this instance.

South Africa: Mr Ken Terry the head of the NDMC indicated knowledge of MDGs, in particular MDG 6. Their successes and strategies, in dealing with this MDG, were:

- effective disaster response coordination
- contingency plans per hazard in the process of being developed
- integrated disaster response planning with the Department of Health (DoH).

The challenge with this is that the DoH has not developed a disaster management plan for the health ministry in particular, and that no meetings with MDG’s on the agenda were attended by the NDMC representatives. The only involvement of the NDMC was that of participating in awareness programs for halting communicable diseases at local, District and Provincial levels. The specific diseases in which they were strongly involved included cholera, Ebola and H1N1. With regards to rating themselves in terms of how far they have gone in achieving the targets in South Africa as a whole from a scale of 1% – 100%, they rate themselves at 60%. This is because of the strong role that disaster management plays in coordinating and supporting the relevant sectors in achieving the targets.

Seychelles: The representative of disaster management in this country, Mr Paul Labaleine, stated that they were aware of the MDGs, and he clearly gave the accurate description of MDG 6. As a country, they explained the following:

‘The Seychelles government is successfully providing free medical care for all Seychellois and residents, free of charge. This included anti-retroviral treatment. The government has also ensured tap water is safe to drink and invest in proper sewerage systems to ensure hygiene and sanitation standards in Seychelles are on par with international standards.’

On the issue of the disaster management office participating in meetings that included MDGs as part of the agenda the respondent replied: ‘Yes. Our office has been involved in the preparation of the National report for the 3^rd^ International Conference on Small Island Developing States’.

## Findings: General discussion of statutes reviewed and interviews

As statutes are formally written enactments of a state or ministry for the purposes of prohibiting, declaring and commanding some action, it was significant for this study to review the SADC countries disaster management statutes in relation to this study. As mentioned earlier this achievement, of MDG’s, requires national legal documents to support the initiative and share the same goals, hence the review of these statutes. All nine countries whose statutes were reviewed, made mention of health care issues and institutions, which was considered an acknowledgement and recognition of disaster management’s close relations with other sectors and disciplines at both national and international level.

The mentioning of diseases, in particular HIV and AIDS, is considered a possible cause of concern for disaster managers. South African statutes contained the richest information sought after in this research. The mentioning of nurses in the Namibian statute was a unique highlight, compared to other statutes, because nurses are considered as primary health caregivers when it comes to dealing with diseases. In Lesotho, despite the declaration of HIV and AIDS as a disaster which was supposed to be a positive move in terms of obtaining international intervention, achieving the targets seems to be a challenge because of the socio-political and economic situation. The unavailability of documents on the internet, or those not in English, can make it difficult for humanitarian international investors to provide humanitarian assistance as statutes are very important for any intervention.

The initial response, from the South African National Disaster Management Centre, before the questionnaire was filled in by its Head, was that the disaster management centres are not involved in or deal with any issues related specifically to the health issues of the millennium development goals. A representative from Lesotho also made the same inference. Malawi’s response to the questionnaire and findings in the statute analysis seems positive in terms of working within their mandate and their willingness to halt diseases. Given the responses from the Zimbabwean, Lesotho and South African officials, it can be assumed that the disaster management statutes are just bare documents created merely to fit in line with national government and international requirements. It should be a prerequisite for anyone, despite the directorate within the disaster management centre, to have comprehensive knowledge about the statutes that govern their operations. Besides the lack of knowledge about these statutes, disaster managers are supposed to lead and coordinate different disciplines and sectors to reduce risks and manage possible disastrous events, and definitely their involvement in all MDGs should not go unnoticed.

### Recommendations

- Most of the countries statutes do not make any reference to international initiatives, health institutions, HIV and AIDS and other diseases, and also most interview responses indicate that the centres are not particularly involved in the achievement of MDGs. It is, thus, imperative for the Millennium Development Goal Post 2015 Development Agenda to make it mandatory for signatory countries to send their disaster management officials to form part of this delegation as they are the main coordinators of humanitarian work in the relevant countries.
- Education, Training and Research, within the field of disaster risk reduction and management at a national and international level, should be compulsory for disaster management officials employed at national disaster management centres. Knowledge and participation in evolving trends within this field is vital.
- SADC offices should create a comprehensive, updated database of resources and documents for research by providing an archive which has all disaster management statutes in English, as it is the main commercial language in the SADC region. It will also be valuable if all national disaster management centres submit their documents to the SADC office.
- A regional approach to managing risk is essential hence national government disaster managers in member states of the SADC should form a well-coordinated, integrated, institutional protocol with a focus on coordination to deal with MDGs and other initiatives.

## Conclusion

The formulation of the MDGs was an innovative initiative that was established to fight poverty and calamities. With such an initiative the study aimed at investigating the involvement and contributions made by the disaster management fraternity since the establishment of this Declaration in the year 2000. This was accomplished through the analysis of national disaster management statutes, the review of literature and telephonic interviews with heads of disaster management centres. Telephonic interviews and the obtaining of statutes proved to be the most difficult method of data collection, especially considering that not all countries disaster management plans are well established. The analysis of some of the obtained statutes did indicate that they embraced the support to fight and contain diseases. However, the disaster management’s role of coordinating humanitarian activities is clearly visible through the statutes analysed, but now the achievability of the specific goal depends on social, political and economic dynamics of the individual SADC states. Further research on the assessment of the SADC health ministries or departments, in the achievement of the sustainable development goals, with a specific focus on health issues, is strongly recommended.
